# Digital education and exercise therapy versus minimal intervention for young people at high risk of early onset knee osteoarthritis after ACL reconstruction: a study protocol for the Stop OsteoARthritis (SOAR) randomized controlled trial

**DOI:** 10.1186/s13063-025-08896-6

**Published:** 2025-06-20

**Authors:** Jackie L. Whittaker, Amanda Cammalleri, Candice Archibald, Alexandra Brooks-Hill, Patrick Chin, Sarah Clark, Jennifer C. Davis, Alison M. Hoens, Michael A. Hunt, Karin Kausky, Angelina Ko, Lise Leveille, Linda C. Li, Parth Lodhia, Justin M. Losciale, Maxi Miciak, Amber D. Mosewich, Adnan Sheikh, Trish Silvester-Lee, Linda K. Truong, Dominic Wade, David R. Wilson, Hui Xie, Xian He Yan, Ewa M. Roos

**Affiliations:** 1 Department of Physical Therapy, Faculty of Medicine, University of British Columbia, Vancouver, Canada; 2 Arthritis Research Canada, Vancouver, Canada; 3 Centre for Aging SMART, University of British Columbia, Vancouver, Canada; 4 Kootenay Therapy Center, Cranbrook, Canada; 5 Department of Orthopaedics, Faculty of Medicine, University of British Columbia, Vancouver, Canada; 6 Sea to Sky Orthopaedics, Whistler, Canada; 7 Allan McGavin Sports Medicine Clinics, Vancouver, Canada; 8 Faculty of Management, University of British Columbia, Kelowna, Canada; 9 Whistler 360, Whistler, Canada; 10 Do Well Therapy, Vancouver, Canada; 11 BC Children’s Hospital, Vancouver, Canada; 12 Fraser Orthopaedic Institute, New Westminster, Canada; 13 Informatice, Decision-Enhancement and Analytic Science Centre of Innovation, George E. Whalen Veteran Affairs Medical Centre, Salt Lake City, USA; 14 Faculty of Rehabilitation Medicine, University of Alberta, Edmonton, Canada; 15 Faculty of Kinesiology, Sport, and Recreation, University of Alberta, Edmonton, Canada; 16 Department of Radiology, Faculty of Medicine, University of British Columbia, Vancouver, Canada; 17 Nexus Rehabilitation, Coquitlam, Canada; 18 Department of Mechanical Engineering, Faculty of Applied Science, University of British Columbia, Vancouver, Canada; 19 Faculty of Health Sciences, Simon Fraser University, Burnaby, Canada; 20 Department of Musculoskeletal Function and Physiotherapy, University of Southern Denmark, Odense, Denmark

**Keywords:** Action planning, Education, Exercise therapy, Goal-setting, Physical therapy, Prevention, Post-traumatic osteoarthritis, Self-management, Telerehabilitation

## Abstract

**Background:**

People who tear their anterior cruciate ligament and have reconstruction surgery (ACLR) are at elevated risk of inactivity, obesity, and early-onset knee osteoarthritis. Consensus recommendations to prevent post-traumatic knee osteoarthritis include person-centered education and exercise-based treatments. The effectiveness of these recommendations is unknown. This study will assess if a digital education and exercise therapy intervention is superior to minimal intervention for improving knee-related symptoms, function, and quality of life in young people after ACLR.

**Methods:**

The Stop OsteoARthritis (SOAR) study is a parallel, two-arm, assessor-blinded, superiority, hybrid effectiveness-implementation type 1 randomized controlled trial. After baseline testing, 166 participants aged 16–35 years, 9–36 months past, a first-time ACLR with ongoing symptoms will be randomly allocated to one of two treatment groups (1:1 ratio, stratified by sex). Ongoing symptoms will be defined as not meeting a Patient Acceptable Symptom State (PASS) on the averaged Knee injury and Osteoarthritis Outcome Score pain, symptoms function in sport and recreation, and quality-of-life subscales (KOOS_4_ < 79). Participants randomized to the experimental intervention will receive a digital (remote videoconferencing) 6-month program of group-based learning, individualized weekly home-based exercise therapy and physical activity program with tracking, and 1:1 physiotherapist-guided counseling. Participants randomized to the minimal intervention control group will receive an educational recording, best-practice guide for ACLR rehabilitation, one videoconferencing session, and tracking. The primary effectiveness outcome is the between-group difference in KOOS_4_ change from baseline to 6-months, with secondary endpoints at 12- and 24months. Secondary effectiveness outcomes include differences in the change of individual KOOS subscale scores, proportions of participants achieving KOOS subscale PASS scores, perceived self-management, and MRI features of knee OA. We will also assess secondary implementation (perceived barriers and facilitators of SOAR delivery), secondary efficiency (incremental cost-utility ratio), and exploratory outcomes. Missing data will be imputed and blinded intention-to-treat analyses performed.

**Discussion:**

By assessing the effect, implementation, and efficiency of a digital education and exercise-based intervention designed to improve the knee health of young people at increased risk of knee osteoarthritis, this study will provide a basis for future scale-up to help curb the mounting burden of osteoarthritis.

**Trial registration:**

ClinicalTrials.gov NCT06195423. Registered on December 22, 2023.

**Supplementary Information:**

The online version contains supplementary material available at 10.1186/s13063-025-08896-6.

## Introduction

### Background and rationale {6a}

Globally, approximately 600 million people live with osteoarthritis (OA) [1, 2]. OA is one of the fastest growing health conditions, a leading contributor to years lived with disability (YLD) [3, 4], and a source of enormous economic burden [5–8]. The rise of OA is driven by an aging population and increased incidence of risk factors—joint injury, inactivity, and obesity [9, 10]. Knee OA accounts for 80% of the burden (15% greater in females) [1, 11]. Currently, no disease-modifying treatments exist [10].

While OA typically affects older adults, the prevalence in younger adults is growing, with a 66% surge in cases of people aged 30–40 years from 1990 to 2019 [11]. In Canada, 1 in 3 people living with OA are diagnosed before age 45 [12], including 50% of the 700,000 youth who hurt their knees each year playing sports [13, 14]. These “young people with old knees” [15] face pain and functional loss for most of their adult lives, leading to inactivity, many YLD, reduced quality of life (QOL) and elevated premature all-cause mortality [14, 16]. As young adults have work and family obligations, this represents a daunting personal and societal burden [14, 17].

People who tear their anterior cruciate ligament (ACL) and undergo reconstruction surgery (ACLR) are at high risk of knee pain and joint deterioration [18]. They are also at 7.5 times greater odds of structural OA within 10 years than their uninjured peers [19]. ACL tears are common (two to six times more in females) [20], on the rise [21], and easy to identify [22]. Despite an urgent need to alter the trajectory towards OA, this group rarely seeks or receives care that promotes OA risk awareness and self-management [23–27], or targets risk factors (muscle weakness, inactivity, adiposity) [10, 18, 28–34].

In the absence of disease-modifying treatments, preventative healthcare is vital for combatting the burden of OA. Primary prevention (reduce risk factor exposure) has proven elusive [35], so strategies that halt, delay, or reduce symptomatic OA severity after risk factor exposure (secondary prevention) are urgently needed [10, 36, 37]. Knowing ACL tears precipitate knee OA presents a unique secondary prevention opportunity. Capitalizing on this requires consensus about when and how to intervene and what outcomes to monitor, which, until recently, had been lacking.

In 2022, consensus recommendations for secondary prevention of post-traumatic knee OA were published [38]. Informed by 7 systematic reviews [14, 19, 39–43], this guidance advocates for person-centered [44] education and exercise therapy targeting self-management [45] and risk factors for re-injury and OA (muscle weakness [28, 46], inactivity [31], adiposity [34, 38]. Ideally, care would start within 3 years of injury and continue across the lifespan. Priority outcomes include pain, function, and quality of life [38], given their link to health-seeking behaviors and mortality [10, 16, 47]. Articular cartilage structure, assessed by MRI, is also recognized as vital for early OA staging, despite conflicting associations with pain [48–50].

Informed by the recommendations [38], past research [18, 24, 29, 33, 51], and end-users, the evidence-based Stop OsteoARthritis (SOAR) program has shown feasibility and good potential to reduce pain and promote self-management and exercise behavior in young persons at high risk of early-onset post-traumatic OA [27, 52–55].

### Objectives {7}

The aim of this randomized controlled trial (RCT) is to assess the effectiveness, implementation, and efficiency [56] of a digital 6-month education and exercise therapy program (SOAR) versus a minimal intervention control for people aged 16–35 years at risk of early-onset knee OA due to a first-time ACLR. The primary objective is to assess the average effectiveness of the SOAR program on an aggregate outcome of self-reported knee pain, symptoms, function, and quality-of-life (mean score of the Knee injury and OA Outcome score, pain, other symptoms, function in sport and recreation and quality-of-life subscales; KOOS_4_) [57, 58] of people at risk of early-onset post-traumatic knee OA compared to a minimal intervention at 6 months, with additional follow-up at 12 and 24 months. We hypothesize that, on average, the SOAR program will produce greater improvements in knee-related pain, symptoms, function, and QOL after 6 months (primary end point) and long-term (12- and 24-month secondary endpoints) compared to the minimal intervention control group. Secondary effectiveness (6-, 12- and 24-month between-group difference in change and patient acceptable symptom states (PASS) for symptoms, function and QOL; change in perceived self-management; and 12- and 24-month change in MRI features of knee OA [59] and early cartilage degradation [60]), implementation (provider and participant perceived barriers and facilitators for SOAR delivery) and efficiency (incremental cost-utility ratio [61]) outcomes will also be assessed. Finally, we will explore the effect of the SOAR program on 6-, 12-, and 24-month changes in patient-specific function, knee-related self-efficacy, fear of movement and re-injury, knee extensor and flexor muscle strength, physical activity, adiposity, health-related QOL, and cartilage composition (MRI T2 mapping), and describe features of physiotherapist counseling that promote self-management, program delivery costs, and healthcare resource use.

## Methods

### Study design {8}

The SOAR trial is a parallel, two-arm (1:1 allocation ratio), assessor-blinded, superiority, hybrid effectiveness-implementation type 1 RCT [62] with embedded cost-utility analyses and 1:1 interviews informed by the Standard Protocol Items: Recommendations for Interventional Trials statement (SPIRIT; see Supplementary Material) [63]. Reporting of the RCT will conform with the Consolidated Standards of Reporting Trials statement for reported RCTs (CONSORT) [64–66], Template for Intervention Description Replication (TIDieR), Consensus on Exercise Reporting Template (CERT), Reporting of Control Interventions in Efficacy Trials of Physical, Psychological and Self-Management Therapies (CoPPS) [67], Consolidated Health Economic Evaluation Reporting Standards (CHEERS) [68], and Consolidated Criteria for Reporting Qualitative Studies (COREQ) [69]. Multi-focus designs (effectiveness, implementation and efficiency) can accelerate research translation to real-world settings. A type 1 hybrid effectiveness-implementation RCT has a main focus of assessing intervention effectiveness and a secondary focus of understanding implementation context [62]. This design is appropriate as we have shown feasibility [53] and indirect evidence of SOAR effect [54], the intervention is minimal risk [53, 70], and there are no fully powered superiority trials to inform non-inferiority or equivalence designs [71]. Table 1 provides an overview of the study design.

Table 1 Schedule of enrollment, interventions and assessments.

**Table 1:**
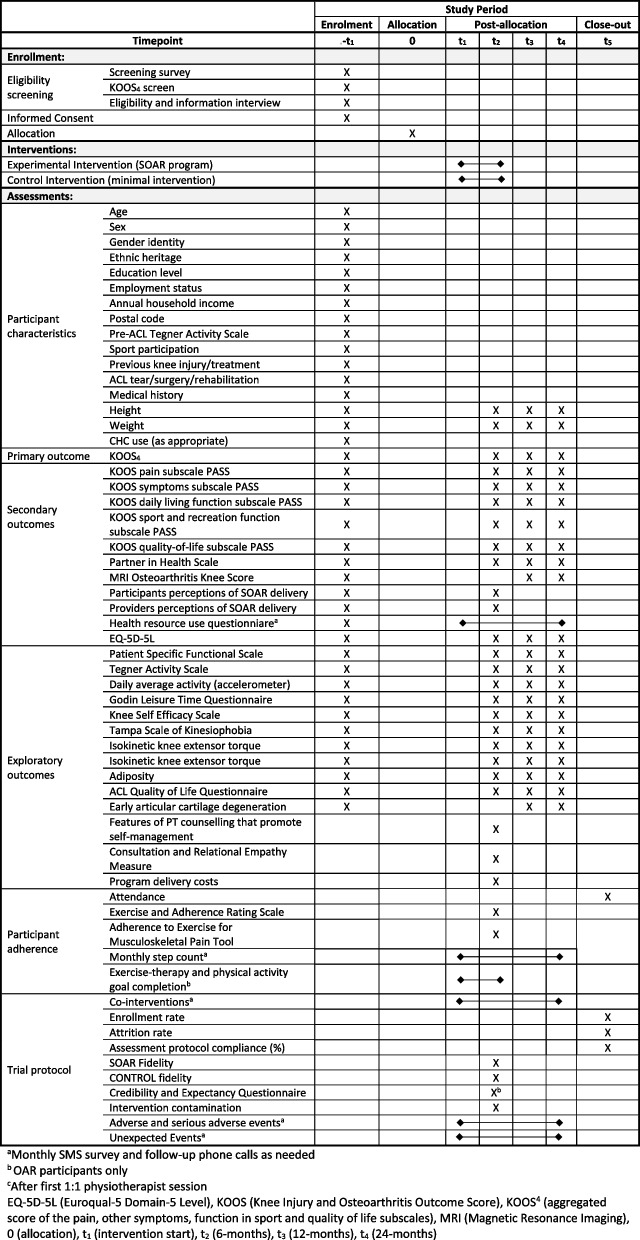
Schedule of enrollment, interventions and assessments

### Study setting {9}

The study will be conducted at the University of British Columbia (UBC) and Arthritis Research Canada (ARC), Vancouver, Canada. Participants will be recruited from collaborating orthopedic surgeons, public hospitals and sport and exercise medicine physicians, and physiotherapy clinics in the greater Vancouver region. In-person data collection will take place at UBC-Point Grey Vancouver Campus. Interventions will be delivered through an online videoconferencing platform (UBC hosted Zoom©).

### Participants {10}

One hundred and sixty-six participants meeting the eligibility criteria (Table 2) will be included.

**Table 2:** Eligibility criteria

Inclusion criteria	Exclusion criteria
• Age 16–35 years (high incidence of ACL tear) [72] • 9–36 months past a first time ACLR [13, 18, 26, 73] • Score below a KOOS_4_ PASS (< 79 points) [38] • Not receiving knee care from a health or fitness provider • No scheduled surgical procedures that would interfere with exercise • Currently live in British Columbia, Canada and willing to attend 4 in-person testing sessions • Have daily access to an email address and a computer with internet • Willing to wear an activity tracker for the study	• Unable to communicate in English • Past diagnostic imaging confirmed ACL tear (complete or partial), meniscal tear, or intra-articular fracture to the ACLR knee • Past physician diagnosis of knee OA • Inflammatory arthritis or other systemic conditions • Lower limb injury, surgery, or intra-articular injection in the past 6 months • Currently pregnant • MRI contraindications^a^

*ACL* Anterior cruciate ligament, *ACLR* Anterior cruciate ligament reconstruction, *KOOS*_4_ mean of the Knee injury and Osteoarthritis Outcome Score pain other symptoms, function in sport and recreation and quality-of-life subscales, *OA* Osteoarthritis, *PASS* Patient acceptable symptom state

^a^Body weight > 400 lbs, pacemaker or any other implanted medical device, brain or ferromagnetic aneurysm clip, metallic prostheses or shrapnel, bullets or other metal fragments, surgery, medical procedure, or tattoos in the last 6 weeks

### Sample size {14}

The study sample size is based on a primary analysis of superiority of the 6-month KOOS_4_ response to the SOAR program considering pilot RCT data [54], systematic review of meaningful KOOS_4_ thresholds in the study population [42] and typical ACLR and knee OA exercise programs effect sizes (0.45–0.50) [40, 74]. Using a KOOS_4_ response standard deviation of 21 points (pilot RCT), α = 0.05 and 1 − ß = 90%, 66 people/group (regression model with 4 covariates and combined *r*^2^ = 0.3) are needed to detect a mean between group KOOS_4_ minimal important difference (10 points) [42], which is consistent with a 0.476 effect size. A sample of 166 (83/group) allows for 20% attrition. Calculations were done with the analysis of covariance procedure in Power Analysis and Sample Size 13 (NCSS, USA) [75].

### Recruitment {15}

Potential participants will be primarily recruited at a post-ACLR orthopedic surgeon visit (approximately 6–12 months post ACLR). We will also leverage sport medicine, physiotherapy, general community (including non-profits offering inclusive sport access) and social media networks. Potential participants will be directed to a short online screening survey, and those potentially eligible will be emailed a study information sheet and unique URL link to an abbreviated KOOS to assess eligibility (KOOS_4_ score). A research coordinator will schedule a telephone/videoconferencing call with potentially eligible individuals to confirm eligibility and answer study questions before sending a unique URL to an online study consent form housed on a secure, UBC-hosted web application designed to support Research Electronic Data Capture (REDCap; see Supplementary Materials) [76, 77]. On enrollment, participants will be assigned a unique anonymous identifier (study ID) that will be used to maintain their confidentiality throughout the trial.

### Allocation {16a, b, c}

After consenting and baseline testing, participants will be randomly assigned to SOAR or control groups in a 1:1 (concealed) allocation ratio, stratified by sex (to enable sex and gender-based analyses) in variable block sizes (to balance group uniformity and reduce sequence predictability). A research coordinator will provide participants with an opaque sealed envelope containing group allocation assigned by a software randomization module (REDCap) [76, 77] with a computer-generated randomized number schedule made by an arms-length statistician. An automated audit trail will track randomization date, stratum, and allocation.

### Blinding and bias {17a, b}

The nature of the interventions does not allow for participant or provider blinding. Participants and providers will be aware they are receiving or delivering one of two knee health programs with varying levels of supervision. To reduce confirmation bias, we will use methods proven in our pilot RCT to blind assessors and data analysts to group allocation. We will reduce contamination bias by ensuring that control physiotherapists are naïve to SOAR, and taking steps to eliminate interaction of SOAR and control participants (informed of study at last surgeon visit; virtual delivery), and SOAR and control physiotherapists (not from same site). A self-reported primary outcome will ensure participants and assessors are blinded to previous scores. To reduce interpretation bias, blinded results (group A vs. group B) will be presented to the research team*,* who will agree on two alternative written interpretations before the biostatistician unblinds the data [78].

### Participant timeline {13}

**Fig. 1:**
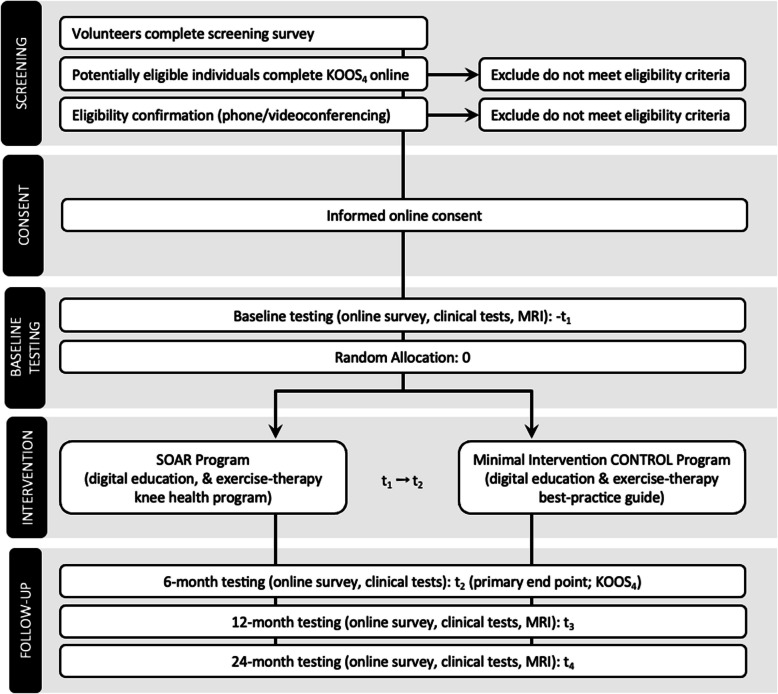
Participant flow chart

Participant study flow is outlined in Fig. 1.

### Interventions {6b, 11a}

An overview of the experimental and control interventions consistent with the TIDieR guidelines is provided in Table 3. After the baseline assessment, participants will receive their intervention specific workbook (in the sealed opaque envelope containing their group allocation), a wrist-worn activity tracker (Fitbit® Inspire 3, Fitbit Inc, San Francisco, CA, USA) and a resistance loop set (Chimaera®) providing up to 100 lbs of load to enable exercise progression. After agreeing to Fitbit’s® privacy policy, participants will be oriented to the device, instructed to wear it 24 h per day, and share their Fitbit® activity “Dashboard” with researchers. Participants will be asked to refrain from introducing new knee-related treatments (health or fitness provider) beyond the interventions they are assigned. Any co-interventions will be captured with the Health Resource Utilization Questionnaire (HRU; see below).

**Table 3:** Overview of interventions according to the TIDieR guideline

Brief name	SOAR program (intervention)	Minimal intervention control (control)
Why	• Evidence-based consensus recommendations to optimize knee health after injury include person-centered education and exercise therapy targeting self-management and risk factors for re-injury and osteoarthritis (muscle weakness, inactivity, adiposity) • Patients identify a “post-ACLR rehabilitation” care gap related to learning how to self-manage their long-term knee health	• Offer evidence-based principles for ACLR rehabilitation and knee health but is inert of tailoring, goal setting, intentional and reflective self-monitoring, or performance feedback • Provide an expectation of therapeutic benefit, and control for natural history, regression to the mean, and contextual effects
What materials	• Fitbit Inspire® activity tracker (Google LLC) • Resistance loop set (Chimaera®) providing up to 100 pounds of resistance • Workbook: study and program overview, educational material, instructions for rating perceived effort, SMART goal setting, Fitbit use, and exercise log • Exercise videos: videos of example ACLR exercises and exercise classes that participants can use for their SMART goals and to supplement their home program	• Fitbit Inspire® activity tracker (Google LLC) • Resistance loop set (Chimaera®) providing up to 100 pounds of resistance • Best-practice workbook: study overview, instructions for rating perceived effort, setting generic goals, Fitbit use, example ACLR exercises (no prescription), and exercise log • Educational video: knee anatomy, ACLR and broad goal setting and exercise principles
What procedure	1. Fitbit®: Participants are encouraged to wear 24 h a day for the duration of the study 2. Virtual group education: what is OA, how to reduce OA risk and SOAR program overview 3. Virtual knee exam: Participants and PTs co-identify individual residual functional deficits 4. Virtual 1:1 PT-guided counseling: Using a BAP approach PTs guide participants to identify ≥ 1 home-based exercise therapy and ≥ 1 physical activity SMART goal for week 1. Goals are modified until participant’s confidence to execute rates ≥ 7/10. Actions to address perceived barriers are discussed 5. Weekly home-based exercise therapy, physical activity, with activity tracking: Participants towards their tailored SMART goals. Exercise and activity completion, dose, and any pain are logged 6. Virtual 1:1 PT-guided counseling sessions: Using a BAP approach, PTs guide participants to progressively add and modify tailored exercise therapy and physical activity goals based on the past weeks goal completion, activity (Fitbit®), symptoms and obstacles	1. Fitbit®: Participants are encouraged to wear 24 h a day for the duration of the study 2. Review best-practice workbook 3. View educational video: Contains sufficient information to develop a best-practice exercise program 4. Participants are expected to exercise unsupervised using the information and materials provided 5. Virtual PT session: One session with a PT (naïve to BAP and SOAR) where participants can ask questions and seek clarification about their Fitbit®, or content of the educational video or best practice workbook
Who provided	• Research staff: Assist with to Fitbit® set-up • Registered MSK PTs (BAP certified^a^ and SOAR trained^b^): Interactive education session, knee exam, 1:1 counseling sessions and recorded group classes	• Research staff: Assist with to Fitbit® set-up • Registered MSK PTs^c^ (naïve to SOAR and BAP): Educational video and virtual PT session
How	• Virtual delivery (videoconferencing-Zoom®) and recorded videos (secure server) • Education classes: group setting • Fitbit® set-up, knee assessment, PT counseling: individually • Home-based program and exercise videos: individual and unsupervised	• Virtual delivery (videoconferencing-Zoom®) and recorded videos (secure server) • Educational video: individually, unlimited unsupervised • Fitbit® set-up, PT session: individually • Home-based program: individual and unsupervised
Where	Device (computer, laptop, or tablet) with internet access	
When and how much	• Group education: 1 h • Knee assessment: ½ h • 1 st PT-guided counseling session: ½ h • Home-based program: 24-week program of ≥ 1 exercise therapy and ≥ 1 physical activity SMART goal with of a task and adequate dose (target RPE of 6–8 or 6–8 RIR) tailored to address the participants functional limits and goals. Number of goals will vary by week and participant based on tailoring. Exercise dose (sets, repetitions, RPE, or RIR) and goal completion will be logged. Fitbit® wirelessly synchronizes with an online “Dashboard.” • Weekly PT-guided counseling sessions: one weekly ≤ ½-h session for 16 weeks (weeks 1–16), and bi-weekly for 8 weeks (weeks 17–24)	• Educational video: ½ h (unlimited) • Best-practice workbook: unlimited • PT session: one ≤ 45-min session
Tailoring	• Knee Exam: Participants and PTs will co-identify and prioritize participants unique knee-related functional deficits and goals • Program: Participants will be guided to identify exercise therapy and physical activity goals (task and dose) to address their unique functional deficits/needs. Goals will be added and modified based on the past weeks goal completion, physical activity (Fitbit®), symptoms and individual obstacles	• No tailoring
Modifications	There will be no modification of the intervention during the study	
How well (planned)	• Participant adherence: attendance, proportion of partial and full exercise therapy and physical activity goal completion, adherence behavior (EARS, ATEMPT), and monthly step count • PT fidelity: 30-item checklist applied to all recorded 1:1 sessions for 5 random participants/PT • Participant perception of credibility and therapeutic benefit will be assessed with the CEQ after their first 1:1 PT session	• Participant adherence: attendance, adherence behavior (EARS, ATEMPT), and monthly step count • PT fidelity: 6-item checklist applied to the recorded 1:1 session for 5 random participants/PT • Participant perception of credibility and therapeutic benefit will be assessed with the CEQ after their 1:1 PT session
How well (actual)	This information will be reported in the primary paper	

*ATEMPT* Adherence to Exercise for Musculoskeletal Pain Tool, *BAP* Brief Action Planning, *CEQ* Credibility and Expectancy Questionnaire, *EARS* Exercise Adherence Rating Scale, *MSK* musculoskeletal, *NRS* numerical rating scale, *PT* physiotherapist, *RCT* randomized controlled trial, *RIR* repetitions in reserve, *RPE* rate of perceived exertion, *SMART* specific, measurable, attainable, relevant, and time-bound

^a^BAP certification: complete online modules (5 h) and role-playing scenarios with feedback (3 h), and 1:1 role-playing evaluation

^b^SOAR training: orientation to the program and educational content, simulated virtual knee exam, and SMART goal setting with group debrief, Zoom® orientation, best practice for virtual rehabilitation, research ethics principles, and online PT tracking forms

^c^Control MSK PTs complete training to be able to explain and answer questions about the best-practice guide and educational video without volunteering information beyond the scope of the booklet or video They are also orientated to Zoom® and review research ethics principles

#### Experimental intervention (SOAR program)

SOAR is a 6-month, digital (remote videoconferencing delivery*)*, physiotherapist-guided knee health program that promotes exercise behavior and self-management ability. SOAR was designed iteratively with a complex intervention framework [79] and reflects consensus recommendations [38] and principles that promote positive healthcare experiences and meaningful outcomes (person-centered care [44], shared decision-making [80], behavior change theory [81]). The rationale for physiotherapist delivery is patient preference and scope for safe and effective knee injury exercise prescription [40, 74]. The rationale for virtual delivery is access [82], scheduling flexibility [83], enhanced patient-physiotherapist relationship through continuity and consistency of care [55, 84], and cost-effectiveness [85]. Virtual physiotherapist exam and treatment do not differ from in-person methods for pain, function, and quality-of-life outcomes [86, 87]. Prior to beginning the SOAR program, participants are provided with a workbook containing an overview of the study and SOAR program, educational materials, instructions for rating perceived effort, SMART (specific, measurable, attainable, relevant, time-bound) goal setting, Fitbit use, and exercise log.

SOAR has three components: one-time Knee Camp, tailored weekly home-based program with tracking, and weekly 1:1 physiotherapist-guided counseling. Participants will be randomly allocated to 1 of 4 independent musculoskeletal physiotherapists (licenced, certified in Brief Action Planning [88], and trained and delivered SOAR in our pilot RCT [54]).

Knee camp: This 2-h session involves interactive group education, 1:1 knee exam, and 1:1 exercise therapy and activity counseling. Education topics are patient partner approved, consistent with shared decision-making [89], practice guidelines [90], and programs promoting self-management [91]. During the knee exam, participants and physiotherapists will co-identify and prioritize individual residual functional deficits. Exercise therapy and activity counseling will follow a motivational interviewing Brief Action Planning approach [88]. Physiotherapists will guide participants to identify at least one home-based exercise therapy and one physical activity SMART goal with tasks and adequate dose (target Rating of Perceived Effort) [92, 93] tailored to their unique needs. Goals are modified until participant’s confidence to execute them rates $\ge$ 7/10. Actions to address perceived barriers are discussed.

Weekly home-based program with tracking: Participants will work towards their exercise therapy and activity goals at home. To self-monitor (track) exercise therapy and activity, participants will log exercise completion, Rating of Perceived Effort, and pain (paper or electronic form), and use a commercial wrist activity monitor (Fitbit®, worn 24 h/day) that wirelessly synchronizes to an online dashboard. Exercise logs are a valued active coping strategy after injury [25], and activity trackers provide motivation for activity goals [94, 95]. These data will be used for self-monitoring purposes, to inform weekly physiotherapist-guided counseling sessions, and monitor participant adherence.

Weekly physiotherapist-guided exercise therapy and physical activity counseling sessions: Aligned with patient preferences for regular contact with a licensed clinician with exercise prescription expertise for health conditions [25, 26, 96], participants will attend a 1:1 virtual physiotherapy counseling session (weekly months 0–4, bi-weekly months 5–6)*.* During counseling sessions (~ 15 min), participants and physiotherapists will progressively add and modify personalized SMART exercise therapy and activity goals, considering goal completion, exercise and activity tracking data from the past week, successes, and obstacles encountered [94].

Rationale for SOAR elements: Capability, opportunity, and motivation interact to generate exercise behaviors (COM-B model) [81, 97]. Exercise interventions that incorporate tailoring and the behavioral change techniques of goal setting, intentional and reflective self-monitoring, and performance feedback produced larger effects than those that do not [98, 99]. In the context of SOAR, exercise therapy and activity are the active ingredients for modifying knee health, while other program elements support their execution by promoting psychological capability (education), physical (virtual delivery) and social (alliance with physiotherapist and between participants) opportunity, and automatic (tailored exercise/activity) or reflexive (goal setting, action planning, self-monitoring) motivation [24, 88, 97, 100–103].

#### Comparison intervention

Knowing that the choice of control condition will affect intervention effect size [104], we developed a control minimal intervention with the CoPPS Checklist for Control Intervention and Conduct [67]. The control intervention aims to provide an expectation of therapeutic benefit, while controlling for natural history, regression to the mean, and contextual effects [105], while acknowledging that more elaborate control conditions can mask effectiveness [106].

Control group participants will receive resources to facilitate an unsupervised home exercise program inert of tailoring, goal setting, intentional and reflective self-monitoring, or performance feedback. This includes the same activity tracker and resistance loop set; unlimited access to a 30-min educational video and best-practice workbook (broad exercise principles for ACLR); and 1 virtual session with a control physiotherapist (naïve to SOAR and Brief Action Planning) trained to explain and answer questions about materials but not volunteer further information.

The control intervention replicates many SOAR elements, including materials, provider, delivery method, location, and some (online learning, tracking, physiotherapist interaction), but not all attention (frequency) to ensure it is inert of behavioral change techniques that increase exercise effect. A minimal attention control was chosen over usual care (0.5 knee health visits annually after discharge from ACLR rehabilitation) [23] to reduce overestimating treatment effect; with that said, minimal attention control conditions are common in rehabilitation trials [107–109], enable long-term follow-up, and facilitate data synthesis [107, 110].

### Data collection procedures {11d, 18a, b}

Participants will be evaluated at baseline and 6- (primary endpoint), 12-, and 24-months (see Table 2). At all timepoints, participants will complete patient-reported outcomes using the online REDCap [77] platform and attend an in-person testing session at UBC. At the in-person session, participants will complete a computerized dynamometry assessment of knee muscle function, a bioelectrical impedance scan, and be given a triaxial accelerometer (ActiGraph wGT3X-BT®, Actigraph™, Pensacola, FL, USA) and instructed to wear it for 7 full days before returning it in a pre-paid courier envelope. The option to complete patient-reported outcomes on paper at the in-person sessions will also be offered.

At baseline, 12-, and 24-month follow-up sessions, participants will also attend a 1-hour visit to the MRI Research Centre at UBC, where dedicated technicians will collect MRI studies of the ACLR knee. Healthcare resource use (including co-interventions), monthly step count, and adverse, seriously adverse, and unexpected events will be assessed monthly over the entire study period using a 7-question short message service (SMS) screening survey and follow-up phone calls as appropriate (see below).

### Outcomes {12}

Outcome choice is based on previous research and responsiveness observed during our feasibility and pilot studies [18, 29, 53–55, 111]. Patient and clinician partner guidance about the relevance, meaningfulness, and burden of measures has also been sought. Table 2 provides an overview of the outcomes and timeline of collection.

Baseline characteristics: age, sex, gender, ethnicity, education level, employment status, socioeconomic status (SES; annual household income, postal code), pre-ACL tear activity level, sports participation, and previous knee injury (treatment), ACL tear (concomitant injury/surgery/rehabilitation), and medical history details will be collected [52]. Baseline body mass index (BMI) will be calculated from height (stadiometer) and body mass (Model MC-980U +, Tanita Inc, USA).

#### Primary outcome

##### Aggregate of knee-related pain, symptoms, function in sport and recreation and QOL

The primary outcome is between-group difference in the change in an aggregate measure of knee-related pain, symptoms, function in sport, and QOL from baseline to 6-month follow-up. The KOOS is a valid and reliable patient-reported outcome with sub-scales for pain, other symptoms, daily living function (ADL), sport/recreation function, and QOL across knee injury and OA populations [112]. The KOOS_4_ (average of 4 sub-scales omitting ADL) is a recommended and accepted primary outcome for ACLR RCTs [38, 57, 58, 107] and was responsive in our pilot RCT [54].

#### Secondary outcomes

##### Knee-related pain, symptoms, function in sport and recreation and QOL

To help interpret the clinical relevance of the KOOS_4_, individual sub-scale scores and the percentage of participants exceeding sub-scale PASS [113] scores (pain 89; symptoms 83; ADL 95, sport 72; QoL 73) [38] will be reported by group and timepoint.

#### Perceived self-management

The 12-item Partner in Health Scale (PIH) is a valid and reliable patient-reported outcome measure of self-management across numerous chronic conditions [114, 115] with subscales for knowledge, symptom recognition and management, partner in treatment, and coping [115]. Each subscale item is scored on a 9-point scale, and individual subscale items are summed, with lower scores indicating a better ability to self-manage. The change in PIH between timepoints will be reported.

#### MRI features of osteoarthritis

Knee OA features (e.g., cartilage defects, bone marrow lesions, meniscal tears, osteophytes) on axial, sagittal, and coronal 3D proton-density gradient MRI sequences (3 T scanner, Philips Elition, 8-channel knee coil) will be registered using the valid semi-quantitative MRI OA knee score (MOAKS) [59, 116]. Positioning aids will immobilize the knee to ensure consistent scans [18, 111]. A radiology fellow (supervised by AS) will conduct MOAKS ratings blinded to group allocation. OA feature worsening will be defined as an increase in the size of the lesion using established methods [116]. Changes in MRI features between baseline, 12, and 24 months will be reported.

#### Participant and provider perceived barriers and facilitators of SOAR delivery

On completion of the SOAR program, we will conduct semi-structured 1:1 recorded interviews with all physiotherapists and a purposive maximum variation [117] sample of 15–20 SOAR participants (varied by gender, age, time from ACLR, adherence). Using an inductive approach and interview guide, open-ended queries about SOAR barriers and facilitators will be asked. We will explore therapeutic alliance, goal completion, and self-management with participants, and Brief Action Planning training, and perceived impacts on patient and clinic flow with physiotherapists. We will pilot, then refine interview guides co-developed with patient and physiotherapist partners during data collection to support thorough exploration of emerging themes. Probes/prompts will promote elaboration. Field notes will be taken. Sampling will be informed by ongoing analyses and cease when no new themes arise [117, 118]. Participants will also respond to an online survey where they can anonymously share their satisfaction with the SOAR program.

#### Incremental cost-utility ratio

To assess the value for money (healthcare system perspective) of the SOAR program, we will estimate the incremental cost per quality-adjusted life years (QALY) gained by SOAR versus the control intervention at intervention cessation and end of follow-up. Costs will be estimated with a HRU questionnaire [119], as the study population often uses resources not captured in administrative data [23], and evidence of good agreement between administrative and prospectively collected HRU data [120, 121]. The HRU captures provider visits, hospital admissions, lab and diagnostic tests, and medication use. The HRU will be administered by research staff monthly via a 7-question SMS with phone call follow-up as needed over the study period.

QALYs will be estimated from health-related QOL (EQ-5D-5L) [122, 123] using area under the curve analysis. The EQ-5D-5L (European Quality of Life 5 Dimensions 5 Level) is widely used for cost-utility analyses across multiple countries and conditions [124, 125] (including OA [126, 127]), and assesses five levels across five health domains (anxiety/depression, pain/discomfort, mobility, self-care, usual activities) to produce a health state profile using Canadian conversion tariffs [128].

### Exploratory outcomes

Exploratory outcomes will be reported at all timepoints unless otherwise indicated.

#### Self-reported function

The Patient Specific Functional Scale (PSFS) will be used to assess changes in functional limitations most relevant to participants [129]. The PSFS prompts participants to identify three activities important to them and rate their ability to perform each activity on a 10-point numerical rating scale. Individual scale scores are summed and transformed to a 0–100 scale with higher scores indicating better outcomes. The PSFS is valid and reliable for use in persons with knee injury [129].

#### Knee-specific self-efficacy

The Knee-Specific Self-Efficacy Scale (KSES)[130] is a valid and reliable measure of knee-specific self-efficacy in people with a sport-related knee injury in the previous 5 years [131]. The KSES consists of 22 items organized into two sub-scales (present and future self-efficacy) [130]. Each item is scored on a 0–10-point Likert scale, with higher scores indicating great confidence. Individual item scores are summed to produce a total score. Higher scores indicate higher levels of self-efficacy [130].

#### Fear of movement and re-injury

The Tampa Scale of Kinesiophobia (TSK) is an 11-item questionnaire that measures fear of movement and reinjury [132]. Each item is scored on a Likert scale from strongly disagree to strongly agree. The item scores are summed to produce a total score, with a higher value indicating greater fear. The TSK has evidence of known-groups validity (scores discriminate between athletes who return or did not return to sports participation level after knee injury) [133].

#### ACL-related quality of life

The ACL Quality-of-Life Questionnaire (ACLQOL) is a 32-item questionnaire that measures knee-related quality of life in individuals who have had an ACL tear [134]. The ACLQOL consists of 5 domains related to physical symptoms (5 items), occupation (4 items), recreational activities (12 items), lifestyle (6 items), and social interactions (5 items). Each item is rated on a scale from 0 to 100, and the total score is expressed as an average across domains out of 100%, with higher scores indicating greater knee-related quality of life. The ACLQOL is valid, responsive to change, and has excellent test–retest reliability [134].

#### Self-reported physical activity

The Godin Leisure-Time Exercise Questionnaire (GLTQ) is a 3-item questionnaire that provides a valid and reliable assessment of metabolic equivalents of physical activity during free (or leisure) time [135, 136]. There are established thresholds for sufficient and insufficient leisure time activity based on GLTQ outcomes [135].

#### Daily average moderate to vigorous physical activity

Total daily minutes of moderate to vigorous physical activity will be estimated from a waist-worn tri-axial accelerometer (ActiGraph GT3X®, Actigraph, Pensacola, FL, USA) worn for 7 consecutive days [137]. Participants will wear the device 24 h a day, removing it only for bathing/swimming activities and keep a log of the duration and intensity of non-wear time activities. The average number of 10-min moderate-to-vigorous physical activity bouts [33] over 7 days is a valid and reliable measure of activity in youth and young adult populations [51].

#### Knee extension and flexion strength

Isokinetic peak concentric knee extensor and flexor torque (Nm/kg) will be assessed with a computerized dynamometer (Biodex System 4™, Shirley, NY, USA) at 90°/s over 0–100° of knee motion. The peak gravity-corrected torque reached across 3 repetitions will be recorded and normalized to body weight. These outcomes are reliable, valid, relevant, and recommended measures of muscle function [138].

#### Adiposity

Bioelectrical Impedance (Model MC-980U +, Tanita Inc., USA) is a feasible method for assessing and tracking body composition in clinical settings and will be used to measure total body mass and total fat mass. Bioelectrical impedance has been shown to be valid and reliable in child, youth, and adult populations [139, 140]. The device will be calibrated prior to each scan (according to the manufacturer’s protocol) and fat mass index (FMI) will be calculated as total fat mass (kg) per height (meter) squared.

#### Early knee cartilage degeneration

Preliminary research links exercise therapy to better knee cartilage health in people at risk of post-traumatic OA (post-meniscectomy) [141] and early atraumatic OA up to 12-months [142, 143]. We will provide further insight over 24 months by quantifying changes in cartilage T2 relaxation times (sagittal multi-echo-spin-echo pulse sequences; 12 echoes) a widely used marker of early degeneration [60]. A senior imaging scientist will segment the cartilage manually and calculate average T2 for femoral, tibial, and patellar cartilage plates [144].

#### Therapeutic alliance

The 10-item Consultation and Relational Empathy (CARE) measure will be used to assess the quality of participants’ interactions with their physiotherapist [145, 146]. Each item is scored on a 5-point Likert scale, and items are summed to provide a total out of 50 points, with higher scores indicating greater empathy and interpersonal care. The CARE measure is valid, reliable, responsive, and sensitive [146].

#### Features of physiotherapy counseling that promote self-management

At the end of the 6-month intervention period, semi-structured 1:1 interviews will be conducted with a purposive maximum variation sample (gender, age, adherence, time since injury) [117] of 15–20 SOAR participants. Using video-cued narrative reflection [147], participants will self-select meaningful exchanges with their physiotherapist that promoted self-management while viewing a recording of their 1:1 initial action planning component of knee camp and a random weekly counseling session. Participants will be able to pause and rewind the recording to identify, describe, and elaborate on significant moments, verbal or non-verbal cues, or feelings. A definition of self-management [148] and context-specific examples will be provided. Probes and prompts will provide elaboration. Field notes will be taken, and interviews recorded. Ongoing analyses will inform sampling [117], and data collection will cease when no new themes are identified [118]. Video-recorded healthcare interactions are acceptable and provide valuable practice insights [149]. Video-cued interviews yield rich data as participants simultaneously identify, engage, and reflect on their lived experiences [147].

#### Intervention delivery costs

The cost to deliver the SOAR and minimal intervention control programs will be tracked by research personnel and reported.

#### Participant adherence

We will report intervention attendance, adherence behavior (Exercise Adherence Rating Scale [150] and Adherence to Exercise for Musculoskeletal Pain Tool [151]), and monthly step count (monthly SMS screen and Fitbit®) for both study groups, and percent partial and full exercise therapy and physical activity goal completion for the SOAR group.

#### Trial protocol

Enrollment procedure adherence, enrollment rate (percentage of those eligible who enroll), attrition rate (percentage of those who enroll but do not complete), assessment protocol compliance (percentage complete per follow-up), and adverse and unexpected events will be tracked. We will also assess physiotherapist fidelity (checklists refined in our pilot study applied to all recorded 1:1 sessions for 5 random participants/physiotherapist), intervention contamination (6-month survey question asking participants if they have interacted with anyone in the study receiving the other intervention), and participants’ expectation of benefitting from their assigned intervention (Credibility and Expectancy Questionnaire; CEQ, after the first 1:1 physiotherapist session) [152].

Adverse events will be defined as a health-related event requiring medical treatment, medications, and/or interfering with function for ≥2 days, which do not necessarily have a causal relationship with the treatment or any study intervention. Serious adverse events will include those that result in death, are life-threatening, require inpatient hospitalization or prolong existing hospitalization, result in persistent or significant disability/incapacity, or require intervention to prevent permanent impairment or damage. We will also collate unexpected events (an incident, experience or outcome that is unexpected, puts research participants at a greater risk of harm and may have been caused by the study procedures) [22].

### Stopping rules, intervention discontinuation {11b}

If the planned sample size is not reached within 36 months, we will halt recruitment at 132 participants, which will ensure 90% power to detect a clinically meaningful 10-point difference in KOOS_4_ 6-month response between treatment arms (anticipated 0.476 effect size). An intervention will be discontinued if any adverse event, medical condition, or situation occurs that makes continuing in the study not in a participant’s best interest.

### Data collection management and security {11c, 19}

All patient-reported outcome data will be simultaneously collected and entered using REDCap [76, 77]. Data collected by other electronic means (computerized dynamometry, accelerometer, bioelectrical impedance) will be converted to Microsoft Excel files and uploaded to REDCap using automated data entry tools. Paper data will be entered by a single member of the research team with a research coordinator oversight. The research coordinator will track and audit enrollment, attrition, attendance, study and assessment protocol adherence, and online patient-reported outcomes for completion and quality (i.e., range checks for data values) [77], contact participants to address omissions, and maintain all electronic and paper files.

### Analyses {20a, b, c}

Statistical analyses will be performed by an independent statistician and then evaluated and interpreted in a blinded fashion by the author group (results will be written up as two scenarios; group A having had SOAR and group B having had SOAR) prior to breaking the randomization code [78]. To preserve study integrity and mitigate the risk of type 1 errors and premature conclusions, interim analyses are not planned.

Primary analyses will be *intent-to-treat* (by randomization) and missing data will be addressed as outlined below. We will report descriptive statistics for demographic and possible prognostic variables (socioeconomic status, sport participation, time from injury/ACLR, concomitant injury/surgery, rehabilitation, graft type, reinjury, co-interventions) for all participants, those lost to follow-up, and by study group, and consider or control for group differences when interpreting results.

SOAR superiority will be assessed with a generalized linear mixed regression model (GLMM) for the primary outcome (change in KOOS_4_ from baseline to 6 months) adjusted for continuous [153] baseline KOOS_4_ value, BMI [154, 155], time since ACLR [29], and sex. Given there is a single primary outcome, multiple comparison adjustment is not required. SOAR superiority for secondary outcomes will be assessed with mixed-effect logistic (OA lesion worsening) or linear (individual KOOS subscale scores, KOOS PASS and PIH scores) regression models adjusted for the same variables as the primary GLMM, at stated time points. To gauge the robustness of chosen PASS cut-off scores, we will perform two sensitivity analyses. First, we will consider a cut-point based on the upper bound of the PASS values 95%CI and secondly, a cut-point that would result in 10% more participants in the SOAR group achieving a PASS than the control group; a percentage agreed upon by our clinician partners as sufficient to change their management approach for this patient population considering patients SES, program costs, and the possibility of adverse events.

A strength of GLMMs is that they use all available data by including variables with missing outcome values. The relationship shape between continuous covariates and outcome change will be assessed prior to analyses. Nonlinearities will be addressed via fractional polynomial or restricted cubic spline transformations, as indicated.

Robust statistical methods will be used to address missing data and minimize the potential for skewing results by missing data handling. GLMMs produce valid inference under the missing at random (MAR) assumption (a standard assumption and more plausible than missing completely at random). Missing values are MAR when they can be predicted from observed data (demographic/prognostic variables, outcome values). As recommended, significant demographic/prognostic and outcome variables will be included as model covariates [156]. To reduce bias and minimize statistical power loss, we will use likelihood-based direct estimation to handle missing outcome values (equivalent to imputing missing values infinite times) [157]. We will assess the robustness of estimates to MAR assumption violations with the Index of Sensitivity to Nonignorable missingness (ISNI) using the R package “isni” [158–161]). The ISNI employs selection models for non-random missing data and performs sensitivity analyses to measure any attrition bias arising from uneven study arm follow-up rates. If the missingness in demographic/prognostic variables used in the outcome models is > 5% (reducing proposed GLMM sample size), we will use a distribution-free multiple imputation method [162]. The multiple imputation method imposes no distributional assumptions on missing values, avoids implausible or out-of-range imputed values, and can handle arbitrary missing data patterns. We will examine the missingness profile and imputed values to ensure no multiple imputation bias [163, 164].

To explore the effect of gender identity (woman, man, gender-diverse) and sex (female, male, intersex), all outcomes will be described by treatment group stratified by sex or gender as appropriate, at all time points. Stratified analyses will estimate primary and secondary regression models to explore intervention effect estimates by sex (female, male) or gender (woman, man) to inform future studies. Descriptive statistics will explore differences by cis and diverse gender.

The percentage of physiotherapists achieving 85% or more on the fidelity checklist and checklist items with 70% fidelity or more will be reported. Interview recordings will be transcribed and de-identified. Using a constant comparative approach [118] data will be coded and categories developed by comparing and identifying meaningful patterns across codes. High-order themes will elucidate the relationship between categories. We will look for uniqueness by gender and if found, reanalyse the data with a gender lens. Analysis trustworthiness and credibility will be fostered through data immersion, memoing, reflexive journaling and team discussions. An audit of analytic decisions will be kept [165].

Incremental cost utility ratio will be estimated as ${\mathrm{Cost}}_{\mathrm{SOAR}}-{\mathrm{Cost}}_{\mathrm{CONTROL}}/{\triangle\mathrm{QALY}}_{\mathrm{SOAR}}-{\triangle\mathrm{QALY}}_{\mathrm{CONTROL}}$ for the intervention and post-intervention period.

### Data security {27}

Consent forms and non-identifiable study surveys (questionnaires) will be stored on a secure web application (REDCap) [76, 77] located on a UBC University Data Centre server (Vancouver, Canada) administered by the UBC Advanced Research Computing group in compliance with the BC Freedom of Information and Protection of Privacy Act (BC FIPPA). Physiotherapist Tracking Forms will be housed on the Arthritis Research Canada (ARC) servers, and physiotherapists will be provided a generic ARC email address for communication with participants for the duration of the study. Other study files, including video-recorded interview data or SOAR sessions, and data sets downloaded for analyses will be stored as password-protected computerized files on a secured network server in the Faculty of Medicine at the UBC. UBC and ARC servers are fully secure using 128-bit SSL encryption. Any portable memory devices used for transporting the data will be encrypted and password-protected. Any paper documents will be stored in a locked cabinet at the UBC, which can only be accessed by the Principal Investigator or their delegate (i.e., research coordinator, or research trainee).

### Oversight and monitoring {21a,b, 22, 23, 25, 26}

Protocol modifications will be communicated through updates to the ClinicalTrials.gov registration.

Seriously adverse, adverse, and unexpected events will be monitored by the research coordinator and immediately reported to the Principal Investigator and Trial Data Safety and Monitoring Committee (DSMC) within 7 days as appropriate. Seriously adverse events will also be reported to the UBC Research Ethics Board within 7 days. If an incidental finding is detected in an MRI scan, UBC MRI technologists will notify the Principal Investigator, research coordinator, and study radiologist (AS) using an incidental finding form. The study radiologist will review the MRI scans and provide instructions for follow-up if necessary.

An external researcher with extensive RCT experience will be asked to chair an arms-length DSMC and will independently recruit two to three members (including an external clinical trialist and biostatistician). The DSMC will meet biannually to assess study quality and progress, recommend protocol changes, alert the principal investigator to procedural or ethical issues, and monitor enrollment, randomization fidelity, data accumulation, and serious adverse events. DSMC members will receive study progress and data reports prepared by an external research manager accessing REDCap reports.

### Patient and public involvement

Patient (TSL, AK) and clinician (CA, ABH, SC, PC, KK, LL, PL, DW) involvement has been integral to the development and conduct of this research. Their engagement corresponds to the IAP2 spectrum *collaborate* (partner in all decisions, develop alternatives, identify solutions) [166]. These partners have guided the development and refinement of the SOAR program, feasibility and preliminary efficacy evaluation, outcome choice, and interview materials. They will assist recruitment, data interpretation, and knowledge mobilization activities (identifying/tailoring dissemination messages, invited to co-present/publish), and be engaged with best practice [167].

### Dissemination plans {31}

We have developed a knowledge mobilization plan [168] with BC SUPPORT Unit consultation and patient and clinician partners. Knowledge mobilization goals are to generate and share new knowledge about the effect, implementation, and efficiency of the SOAR program; enhance general population awareness of OA risk; and bridge links with partner groups to facilitate future evaluation, implementation, and scale-up. Knowledge mobilization partners include research groups, patients, clinicians, and knowledge mobilization brokers engaged from study conception to close. Knowledge mobilization messages, based on dissemination science principles [169], will be identified by the full team. These messages will be tailored to specific target audiences and shared through various methods including scientific papers and presentations, research center exchanges, summaries for partner communities, and physiotherapist training modules to support scale-up. Outputs will follow inclusive messaging principles, undergo sensibility checks, and be refined with end-user partners before sharing through users’ preferred mechanisms/networks. Knowledge mobilization impact will be assessed with reach indicators.

## Discussion

The incidence of ACL tears and subsequent ACLR are increasing [170–173], and at least half of these patients will develop future OA along with its associated symptoms, functional limitations, and comorbidities [14, 19, 174]. Given the enormous burden of OA [2], evidence that it is independently associated with reduced time-to-mortality [47], and absence of disease-modifying interventions, strategies that can halt, slow down, or reduce the severity of future OA after an ACLR (secondary prevention) are urgently needed [10].

The current trial will assess the effectiveness, implementation, and efficiency of a digital (remote videoconferencing) physiotherapist-guided education and exercise therapy knee health program designed to improve the knee health of young people at elevated risk of early onset OA after an ACL tear and ACLR.

It builds on a 10-year research program that has assessed the trajectory of OA risk factors after knee injury [29, 51], identified psychosocial factors impacting knee injury recovery [25] and exercise therapy adherence [175], explored exercise priorities after injury [24, 26], established international consensus on how to approach post-traumatic knee OA prevention [14, 19, 38–43], and developed and assessed a novel secondary prevention knee health program (SOAR) [27, 53–55].

Potential limitations and challenges include recruitment, retention (differential loss to follow-up), and participant compliance/adherence. Knowing people are interested in exercise RCTs due to potential benefit and may decline or withdraw if randomized to the “control” arm, leading to finding a smaller or no effect, the RCT is packaged (outward facing materials) as filling a care “void” [27, 176] by comparing two knee health programs with varying levels of supervision. Beyond planned analyses (missing data) we will use proven recruitment (established healthcare provider networks; offer two interventions with perceived benefit that exceed usual care) [177] and retention (set clear expectations before consent; prioritize participants’ queries; provide monthly newsletters, individual knee health reports, $100 CND compensation, Fitbit® and exercise loop set; send multiple appointment reminders using preferred modes; make multiple follow-up testing attempts; use online surveys with clear instructions; offer evening/weekend in-person testing) strategies [178, 179].

Insights gained about the SOAR effect, delivery barriers/facilitators, and possible need for adaptations to support wider implementation [180] will be used to develop physiotherapist e-training modules; inform tailoring for diverse settings (no high-speed internet/technology access, limited digital literacy, cultural safety) [181]; and design future research to assess SOAR reach, effectiveness, adoption, implementation, and maintenance in community settings [182]. This fully powered trial fills a critical void in the care pathway for people who have had an ACL tear/ACLR and represents a vital step in the battle to overcome the ever-expanding burden of OA.

### Trial status

The current protocol is version 1.0, dated January 9, 2024. Recruitment started on May 22, 2024. The estimated completion date is December 30, 2028.

## Supplementary Information

Supplementary Material 1.

## Data Availability

The following study materials can be accessed on the open science framework https://osf.io/dr23e/?view_only=0b3dc80910f045f4b232228d4b999416; Screening survey, Eligibility survey, Eligibility interview script, Study information and consent form, SOAR knee assessment form, SOAR workbook with group education slides, Control workbook with video education slides, SOAR physiotherapist tracking form, SOAR participant exercise log, SOAR fidelity checklist, Adverse events reporting form, and MRI protocol. The final data set containing data from participants consenting to their anonymized data being open access will be made available on Borealis (borealisdata.ca).

## Abbreviations

ACL: Anterior Cruciate Ligament
ACLQOL: Anterior Cruciate Ligament Quality of Life questionnaire
ACLR: Anterior Cruciate Ligament reconstruction surgery
ADL: Activities of daily living
ARC: Arthritis Research Canada
BMI: Body mass index
CARE: Consultation and Relational Empathy measure
CERT: Consensus on Exercise Reporting Template
CHEERS: Consolidated Health Economic Evaluation Reporting Standards
CND: Canadian dollars
COM-B: Capability, Opportunity, Motivation–Behavior
CONSORT: Consolidated Standards of Reporting Trials
CoPPs: Reporting of Control Interventions in Efficacy Trials of Physical, Psychological, and Self-Management Therapies
COREQ: Consolidate Criteria for Reporting Qualitative Studies
DSMC: Data safety and monitoring committee
EQ-5D-5L: Euroqual-5 Domain-5 Level
FMI: Fat mass index
GLTQ: Godin Leisure-Time Exercise questionnare
HRU: Health Resource Utilization questionnaire
KOOS: Knee Injury and Osteoarthritis Outcome Score
KOOS_4_: Aggregated average score of the KOOS pain, other symptoms, function in sport, and quality of life subscales
MOAKS: MRI and Osteoarthritis Knee Score
MRI: Magnetic resonance imaging
Nm: Newton meters
OA: Osteoarthritis
PASS: Patient Acceptable Symptom State
PIH: Partner in Health Scale
PSFS: Patient Specific Functional Scale
QALY: Quality-adjusted life years
QOL: Quality of life
RCT: Randomized controlled trial
REDCap: Research Electronic Data Capture
SES: Socioeconomic status
SPIRIT: Standard Protocol Items: Recommendations for Interventional Trials statement
SMART: Specific, measurable, attainable, relevant, time-bound
SMS: Short messaging system
SOAR: Stop OsteoARthritis
TIDieR: Template for Intervention Description Replication
TSK: Tampa Scale of Kinesiophobia
UBC: The University of British Columbia
URL: Unique remote locator
YLD: Years lived with disability
